# Guided Endodontic Treatment of an Upper Lateral Incisor: A Successful Outcome in the Presence of Procedural Errors—A Case Report

**DOI:** 10.1155/crid/8910494

**Published:** 2025-12-03

**Authors:** Azadeh Hesarkhani, Sahar Molaei, Shiva Tavakkoli Avval

**Affiliations:** ^1^Department of Endodontics, Specialized Endodontic Practice, Tehran, Iran; ^2^Dental and Periodontal Research Center, Tabriz University of Medical Sciences, Tabriz, Iran

**Keywords:** 3D printing, case report, guided endodontics, pulp canal obliteration, root canal treatment

## Abstract

Performing endodontic treatment on calcified root canals is highly challenging and time-intensive. Even with the assistance of a dental operating microscope, the probability of iatrogenic errors is high. The aim of this report is to present successful endodontic treatments for severely calcified maxillary anterior teeth using a 3D endodontic sleeveless guide, providing a basis for upcoming validation through more comprehensive research. A 25-year-old male patient was referred to the dental office with a chief complaint of pain and swelling of his Tooth 2.2 who explained a previous history of trauma. On clinical examination, periapical swelling was observed, and the tooth demonstrated tenderness to percussion and palpation in the periapical region. Radiographic findings included a severely obliterated pulp chamber and a calcified root canal with periapical radiolucency. In order to perform the root canal treatment with an endodontic guide, an optical impression was carried out using an intraoral scanner. Data from the impression and CBCT files were aligned and sent to the dental laboratory. A virtual copy of a drill was superimposed onto the scans in an optimal position that provided access to the root system, using Exoplan software. A digital model was created and exported as an STL file and transferred to a 3D printer to produce a template. Despite several limitations, successful root canal treatment was conducted. At the 6-month follow-up visit, the patient reported no symptoms, and periapical radiography showed a diminished periapical lesion. Static endodontic guidance provides an opportunity to have more reliable and predictable outcomes in treating complex root systems, even in the presence of minor issues such as transportation and file separation.

## 1. Introduction

One of the clinical challenges practitioners may face during their endodontic treatment is pulp canal obliteration (PCO), which frequently complicates both management and prognosis. As reported by the American Association of Endodontists (AAE), this condition, known as calcific metamorphosis (CM), is characterized by the rapid formation of hard tissue within the pulp space, typically as a reaction to trauma, which is classified into moderate to high-risk groups [1], because of the potential for treatment failure and the heightened risk of iatrogenic complications, including perforation or deviation from the primary root canal pathway [2].

On the basis of previous dental literature, PCO has several etiologies. Dental trauma, such as concussion and subluxation, is recognized as one of the most renowned causes leading to PCO [3, 4]. PCO may also develop due to dental treatments (vital pulp therapy, coronal restorations, orthodontic forces, and autotransplantation), systemic challenges (osteogenesis imperfecta, dentin dysplasia, and dentinogenesis imperfecta), various syndromes such as Van der Woude syndrome, and medications like prolonged glucocorticoid intake [5–7]. Additionally, dystrophic calcification in the coronal area and root canal is frequently observed in elderly people, arising from the natural aging process of the pulp complex and a decrease in pulpal blood flow [8].

Clinicians typically postpone the treatment of teeth with PCO until clinical symptoms manifest, including periapical lesions, yellow or grayish discoloration, tenderness to percussion with a periapical index (PAI) score ≥ 3, and a negative response to sensibility testing [5, 9]. Application of cone-beam computed tomography (CBCT), magnification using a microscope, and ultrasonic devices have equipped practitioners to access calcified canals [10–12]. However, since 2016, guided endodontics has incredibly assisted clinicians in the procedure of severely calcified root canal treatment. Currently, several studies have shown its utilization in various fields of endodontics, including endodontic surgery, management of teeth with anomalies such as dentin dysplasia and dens invaginatus, and root canal retreatment of teeth with fiber posts. These studies have predominantly highlighted that the application of guided endodontics noticeably diminishes iatrogenic errors and dentin loss in contrast to those typically seen in traditional canal preparation techniques [13–16]. Guided endodontics is categorized into two types: static and dynamic. Static guides are created by aligning CBCT scans with optical scans of intraoral anatomy or plaster models. Using implant planning/CAD software, virtual directional guides are designed, and then, depth-calibrated dental bur drills are chosen [17]. Following this method for negotiating a calcified canal, the canal can be accessed with greater precision and stability using a drill by creating cylindrical or sleeve-like guides. The smaller inner cylinder, which is made of metal, fits securely into the larger outer cylinder [18]. Sleeveless guides have also been introduced and utilized in some recent studies which have shown that they can be beneficial in patients who have limitations in mouth opening [19, 20].

Dynamic navigation system involves real-time navigation during endodontic procedures via advanced imaging tools. A tracking system integrates CBCT data with optical tracking devices, enabling the clinician to visualize the drill's position and movement on a digital platform throughout the procedure [21].

This report is aimed at presenting successful endodontic treatments for severely calcified maxillary anterior teeth using a 3D endodontic guide without an inner metal sleeve, despite the occurrence of minor complications such as file separation and deviation from the right path. Thus, these findings can provide a basis for future validation through more extensive studies. This case report was written according to the CARE (for CAse REports) guidelines [22].

## 2. Case Presentation

**Figure 1:**
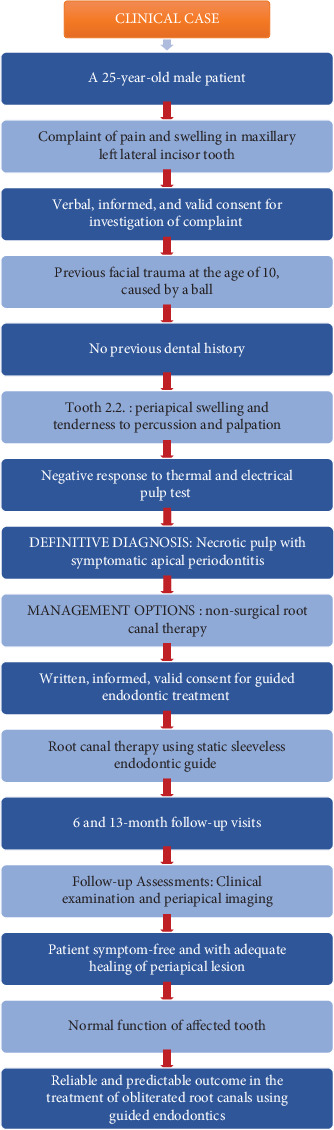
Timeline of events describing case management.

A timeline outlining the key components of case management is presented in Figure 1. A 25-year-old male patient was referred to the dental office in June 2024 with a chief complaint of pain and swelling of his Tooth 2.2. On clinical examination, periapical swelling was observed, and the tooth demonstrated tenderness to percussion and palpation in the periapical region. The patient reported no history of systemic diseases. The medical history revealed a history of facial trauma at the age of 10, caused by a ball, which resulted in soft tissue contusion without any associated bone fracture.

The periapical radiograph of Tooth 2.2 revealed a severely obliterated pulp chamber and a calcified root canal with periapical radiolucency of PAI 5 (a large radiolucent area often indicative of more extensive pathology, such as a radicular cyst or an abscess, with possible involvement of surrounding structures), which confirmed the necessity for CBCT imaging. The final diagnosis for the affected tooth was pulp necrosis following negative results from thermal and electrical pulp testing. Tooth 2.2 was diagnosed as necrotic pulp with symptomatic apical periodontitis. In radiographic terms, the classification of CM was identified as total obliteration. In this case, CM might be a result of a former traumatic injury and was arranged for nonsurgical root canal therapy. According to the AAE, management was deemed to have a high level of treatment difficulty. Therefore, it was decided to perform root canal treatment using a guided template. After consulting with the patient and addressing all of his questions and concerns, consent was obtained for the recommended course of action.

**Figure 2:**
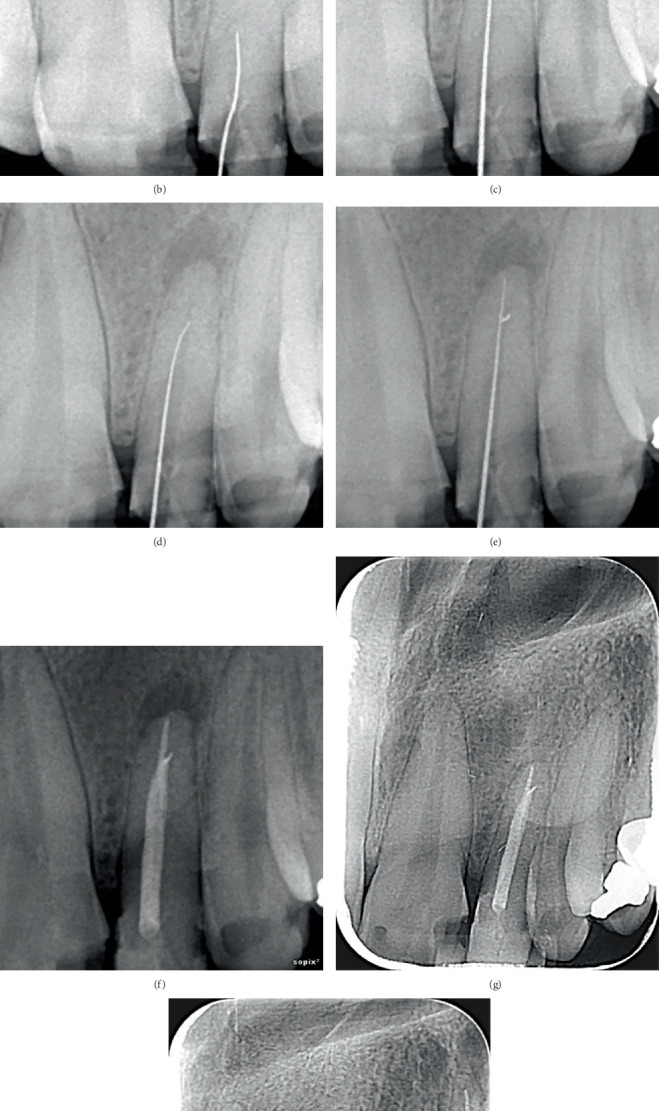
(a) Pretreatment CBCT imaging of the tooth. (b) Minor transportation was observed after initial attempts at root canal negotiation. (c, d) Further attempts to reach the main root pathway. (e) Successful negotiation of the root canal and a remaining separated file. (f) Final obturation of the canal. (g) Periapical radiograph at the 6-month follow-up visit. (h) Periapical radiograph at the 13-month follow-up visit.

**Figure 3:**
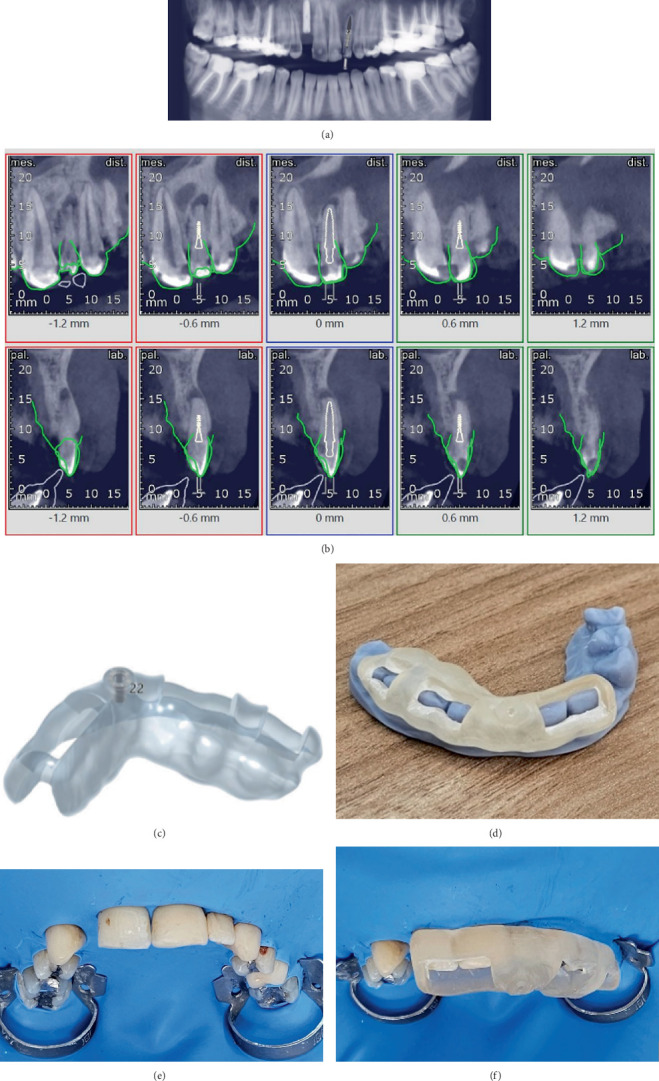
(a, b) Superimposition of drill in order to design the drill path of the endodontic guide in panoramic view and cross-sectional view. (c, d) Model and resin template of the endodontic guide. (e) Multiple-teeth isolation using rubber dam. (f) Placement of the template during the treatment session.

Preoperative high-resolution CBCT (NewTom, Verona, Italy) was performed in order to showcase the zone of obstruction (Figure 2a). The presence of periapical periodontitis was also confirmed via CBCT. Then, an optical impression was taken by an intraoral scanner (Medit i700, Medit Corp., South Korea) to obtain a digital representation of the patient's dental arch. The data from the impression and CBCT files were aligned and sent to the dental laboratory. Exoplan software (Exocad, Version 3.0 Galway) was used to process the data so that the target could be assessed in detail. A virtual copy of a drill with a diameter of 1 mm and a length of 22 mm was superimposed onto the scans in an optimal position, which provided access to the established root system (Figure 3a,b). A digital model was created and exported as an STL file and transferred to a 3D printer (Phrozen, Sonic Mini 8K, Taiwan) to produce a biocompatible resin-based and sleeveless clear template (Figure 3c,d).

During the treatment session, the fit and stability of the guide were checked. Local anesthesia was then carried out via the use of 2% lidocaine with 1:100,000 epinephrine (Darupakhsh, Tehran, Iran) to ensure adequate pain control during the procedure. A multiple-teeth isolation technique was selected. Two clamps were placed on the posterior teeth (Teeth 1.6 and 2.6). A rubber dam (Sanctuary, Medium Size, Malaysia) was carefully passed through the interproximal contacts using dental floss to achieve optimal isolation and adaptation, which in turn allowed the passive fit of the guide. Then, the 3D model was placed and secured with fingers, and the entrance to the access cavity was marked through the sleeve on the surface of the tooth crown (Figure 3e,f). An access cavity was prepared using a dental operating microscope (Sometech, VOMS 100, South Korea) with a diamond access bur (Jota AG, Switzerland) through enamel and a #10 Munce discovery bur (CJM Engineering, Santa Barbara, California, United States) through dentin. Notably, the endodontic guide was modified by slight trimming to allow the bur to penetrate more. A periapical radiograph was obtained during drilling to ensure the correct path toward the root canal. Minor transportation occurred after initial attempts at root canal negotiation (Figure 2b). This was likely due to the type of the endo-guide (sleeveless) and the large size of the hole, which reduced the accuracy. Therefore, the direction of the drill was slightly altered to the mesial part of the tooth. After drilling to the appropriate depth for negotiation, multiple hand K files (#6, #8, and #10) (MANI Inc., Tochigi, Japan) and C files (#8 and #10) (VDW GmbH, Munich, Germany) were used. It is noteworthy that, despite successful negotiation of the canal, a small piece of the separated file was observed in the periapical radiograph, which was the terminal 2-mm segment of a K file #10 (Figure 2e). However, it was effectively bypassed using smaller files and navigating around the separated fragment. After slow and careful advancement, the glide path was created. After canal preparation to Size #25 with a 4% taper using the Rogin rotary file system (Rogin Dental, Shenzhen, China) and thorough 5.25% sodium hypochlorite (NaOCl) (Nik Darman, Tehran, Iran) irrigation, owing to excessive fatigue and the lengthy procedure, intracanal medicament (Nik Darman, Tehran, Iran) was used, and the next steps of treatment were postponed to another session.

At the second appointment, the previous swelling had entirely resolved. Hence, it was confirmed that the right path was negotiated. The complete irrigation protocol for the root canal was performed again to ensure optimal disinfection. The final irrigation protocol included three cycles of 5.25% NaOCl, each activated ultrasonically (Woodpecker, Endo 3, China) for 20 s, followed by 17% EDTA (Nik Darman, Tehran, Iran) for 1 min and 2% chlorhexidine (Nik Darman, Tehran, Iran) to enhance canal cleanliness and remove the smear layer. Then, obturation was performed using the warm vertical condensation technique with a size of 25, 4% gutta-percha (Meta Biomed Co. Ltd., Cheongju, South Korea), and AH Plus Jet sealer (Dentsply Sirona, Bensheim, Germany) (Figure 2f). The patient was arranged for follow-up appointments in order to evaluate the response of the tooth and surrounding structures to the treatment. At the 6 and 13-month follow-up visits, the patient reported no symptoms, and periapical radiography revealed a diminished periapical lesion (Figure 2g,h).

## 3. Discussion

Symptomatic apical periodontitis in PCO cases brings about a significant challenge for dental practitioners. The possibility of iatrogenic errors and excessive loss of dentin may negatively affect the prognosis of treated teeth [2]. Nevertheless, guided endodontics appears to be a unique and secure method for addressing this clinical condition [23]. Numerous studies have investigated the use of guided endodontics for detecting calcified canals. As an example, Haro et al. demonstrated the accuracy of static guides in locating the calcified canal of the lower central incisor, reporting favorable results after 3 years of clinical and radiographic follow-up [24]. Likewise, Torres et al. noted that guided endodontics had significantly better outcomes than did the freehand technique which resulted in fewer technical failures in teeth with PCO and apical periodontitis [25].

A systematic review by Peña-Bengoa et al. related to the effectiveness of guided endodontics in locating calcified root canals revealed that although it is a technique that still presents limitations, guided endodontics provides an opportunity for conservative access, lowers mistakes, and is efficient in locating calcified canals [26]. The use of guided endodontics in similar cases to the present study is vital for enhancing precision, reducing the risk of complications, and ensuring more predictable outcomes. It is particularly valuable in cases with complex or calcified root systems, since it provides the opportunity for safer navigation, reduces the possibility of harm to adjacent structures, and enhances the effectiveness of treatment [27].

Other studies have also introduced innovative static guides, reporting satisfactory results. Studies by Fornara et al. and Wu et al. illustrated that static guides such as titanium-based templates or dice-inspired multifunctional 3D printing guided splints can also be taken into account as treatment enhancers in calcified canals [19, 28].

Even though static guided endodontics offers a dependable and precise procedure, there are notable disadvantages to its application. Some features of guides, such as fixed angulation, size, and depth, are pretty tricky to modify. In addition, production costs and time-consuming planning and manufacturing are among other challenging aspects of this treatment option [29]. Furthermore, the placement of static guides is not feasible in patients who have limited mouth opening or in posterior areas where access is not easy [30]. However, sleeveless guides are able to provide more comfort in patients with improper maximum mouth opening. Torres et al. utilized a sleeveless guide system that navigated the head of the handpiece instead of the drill. The outcomes indicated that this technique appears to be a more dependable option than the classic model of an endodontic guide for negotiating PCO in cases with limited interocclusal space [20]. In our case, we utilized a metal-free sleeveless endodontic guide, which allowed us to modify its height during the procedure by trimming it slightly with a bur, enabling greater bur penetration.

One of the challenges in this case was that the guided template did not have an inner metal sleeve and that the diameter of the guiding hole was large, which caused a slight deviation from the intended path. This highlights the importance of considering the drill and guiding cylinder diameter and its impact on accuracy. Using a smaller drill is able to preserve more tooth tissue, but it can increase the chance of drilling deviations. Larger drills can reduce the risk of errors but may remove more tissue [31]. A more critical evaluation of the drill path technique by Buchgreitz et al. showed that the drill path performance was considerably more precise for mandibular teeth than for maxillary teeth. Nevertheless, they suggested that a slight deviation of the bur can be classified as “acceptable” precision. The “acceptable” definition refers to cases where some deviation was present, but the canal could still be located and prepared with instruments and when follow-up visits indicated signs of a healing cycle [31]. Accordingly, our case showed an acceptable deviation due to successful root canal treatment and the healing of the apical lesion in follow-up.

Another challenge of the present case was the separated file. There are a series of preventive steps the practitioner should follow to avoid file breakage, such as performing a straight line of access into the root canal, preflaring of the coronal section, and choosing the most suitable instruments [32]. Despite carrying out every precautionary stage in this case, a small piece of file was separated. Since attempts for fractured file removal may result in complications including ledge formation, excessive enlargement of the canal, or perforation, the potential risks of file removal should be carefully assessed against the minor advantage [33]. Therefore, the practitioner had decided to bypass the fragment of the file in order not to reduce the strength of the tooth structure.

Dynamic navigation is another type of guided endodontics that provides practitioners with real-time guidance based on customized CBCT data that is used for the positioning of drills. Although professional training is necessary for this technique and requires higher costs, this new technology also improves the accuracy and safety of complex endodontic treatments. One of the major benefits of this method is that any adjustment required by a clinical situation can be incorporated into the working plan at any time during the procedure [30, 34]. Dianat et al. successfully used this method in order to perform root canal therapy for a tooth that had root canal calcification and apical periodontitis. The follow-up sessions showed no undesirable symptoms or signs [35].

Finally, regarding the strengths and weaknesses of static guided treatment options in calcified canals, additional studies should be conducted for similar cases utilizing dynamic navigation to compare the practicability of all approaches among practitioners with different levels of proficiency. Moreover, endodontic guides with smaller guiding holes and metal guiding cylinders are recommended in future studies in order to boost the precision of treatment. Additionally, since most of the available evidence on guided endodontics is derived from case reports, future research should focus on studies with larger sample sizes and controlled designs directly comparing static and dynamic navigation techniques to provide stronger clinical evidence.

## 4. Conclusion

Planning for the starting point of the access cavity via an endodontic guide can be considered a strong alternative in the management of teeth with POC. This technique provides an opportunity to have more reliable and predictable outcomes in the treatment of complex root systems, even in the presence of minor issues such as transportation and file separation.

## Data Availability

The data supporting the findings of this study are available from the corresponding author upon reasonable request.
